# Sestrin2 provides cerebral protection through activation of Nrf2 signaling in microglia following subarachnoid hemorrhage

**DOI:** 10.3389/fimmu.2023.1089576

**Published:** 2023-01-24

**Authors:** Youqing Yang, Han Ding, Chenxing Yang, Jie Wu, Youyuan Bao, Shihai Lan, Lin Zhou, Lu Zhou, Bangliang Liu, Tao Hong, Xichen Wan, Xiao Wu

**Affiliations:** ^1^ Department of Neurosurgery, The First Affiliated Hospital of Nanchang University, Nanchang, China; ^2^ Department of Rehabilitation Medicine, The First Affiliated Hospital of Nanchang University, Nanchang, China; ^3^ Department of Neurosurgery, Shanghai Tenth People’s Hospital, Tongji University School of Medicine, Shanghai, China

**Keywords:** subarachnoid hemorrhage, microglial polarization, sestrin2, neuroinflammation, Nrf2

## Abstract

Subarachnoid hemorrhage (SAH) is a neurological emergency characterized by dysfunctional inflammatory response. However, no effective therapeutic options have been reported so far. Microglia polarization has been proposed to exert an essential role in modulating inflammatory response after SAH. Sestrin2 is a stress response protein. Growing evidence has reported that sestrin2 could inhibit M1 microglia and promote M2 microglia polarization. The current study investigated the effects of sestrin2 on microglia phenotype switching and the subsequent brain injury and sought to elucidate the underlying mechanism. We conducted an endovascular perforation SAH model in mice. It was found that sestrin2 was significantly increased after SAH and was mainly distributed in neurons and microglia. Exogenous recombinant human sestrin2 (rh-sestrin2) evidently alleviated inflammatory insults and oxidative stress, and improved neurofunction after SAH. Moreover, rh-sestrin2 increased M2-like microglia polarization and suppressed the number of M1-like microglia after SAH. The protection by rh-sestrin2 was correlated with the activation of Nrf2 signaling. Nrf2 inhibition by ML385 abated the cerebroprotective effects of rh-sestrin2 against SAH and further manifested M1 microglia polarization. In conclusion, promoting microglia polarization from the M1 to M2 phenotype and inducing Nrf2 signaling might be the major mechanism by which sestrin2 protects against SAH insults. Sestrin2 might be a new molecular target for treating SAH.

## Introduction

Subarachnoid hemorrhage (SAH) is a multiphasic neurological disease with worse prognosis (1, 2). Most SAH survivors suffer permanent neurological disabilities (3). Recently, substantial evidence has indicated that the early brain injury (EBI) is the determinant factor to poor prognosis after SAH (4–6). In the pathological cascade of EBI, the innate inflammatory correlates with unfavorable clinical outcomes in SAH patients (7, 8). However, the effective therapeutic interventions in clinical are still lacking. Therefore, targeting inflammatory response holds great potential for SAH treatment.

Upon the onset of SAH, inflammatory response occurs not only in the brain parenchyma, but also in cortical regions. The infiltration of immune cells, activation of microglia, and reactive oxygen species (ROS) are central to the inflammatory insults after SAH. Among them, microglia activation is crucial for post-SAH neuroinflammation (9–11). Studies have indicated that microglia have a dual role in central nervous system (CNS) diseases. Microglia could polarize into two phenotypes, including M1 phenotype and M2 phenotype. M1-polarized microglia could produce proinflammatory mediators and increase ROS to further aggravate inflammatory insults. In contrast, M2-polarized microglia could release anti-inflammatory agents including IL-10 and IL-13 to reduce inflammation and promote brain repair (12–14). Thus, strategies aiming to inhibit M1 microglia polarization and promote M2 microglia polarization might be a viable treatment for SAH.

Currently, most reported compounds and molecular targets could inhibit M1 microglia polarization. However, few compounds and targets have the ability to promote M2 microglia polarization in acute brain injuries and neurodegenerative diseases. Sestrin2 is a stress response protein. Growing evidence has demonstrated that sestrin2 is involved in modulation of multiple biological functions, such as immune response, oxidative insults, endoplasmic reticulum, ischemia, and hypoxia (15–17). More importantly, recent studies indicate that sestrin2 could inhibit M1 microglia and promote M2 microglia polarization (18). In CNS diseases, sestrin2 has been demonstrated to protect against traumatic brain injury (TBI), ischemic stroke, Parkinson’s disease, and Alzheimer’s disease (19–22). However, less is known regarding the role of sestrin2 on EBI after SAH and the underlying molecular signaling. Therefore, we investigated the influence of sestrin2 on microglia phenotype switching and the subsequent brain injury after SAH and sought to elucidate the underlying mechanism.

## Material and methods

### SAH mice model

Male C57BL/6 (18 wk old, 25–30 g) mice were used in the present study. All experiments were approved by the Ethics Committee of The First Affiliated Hospital of Nanchang University. The endovascular perforation SAH model was conducted according to previous studies (23). In brief, after anesthetization with an intraperitoneal ketamine/xylazine, the carotid artery was exposed. A sharp 6–0 monofilament nylon suture was used to perforate the bifurcation of the anterior and middle cerebral arteries. After the operation, animals were placed into a heating chamber to recover. Sham-treated mice underwent the same procedures without perforating artery vessel. The severity of SAH was evaluated by using a SAH grading system (24). SAH mice that received with a score < 8 were excluded from the current study.

### Experimental groups

In the first experiment, mice were randomly assigned to sham and SAH (6 h, 12 h, 24 h, 48 h, 72 h) groups. Western blot analysis and immunofluorescence staining were employed to explore sestrin2 activation after SAH.

In the second experiment, mice were randomly assigned to sham, SAH, and SAH treated with recombinant human sestrin2 (rh-sestrin2) (1μg, 3μg, and 9μg) groups. Neurological function, ELISA, western blot analysis, and immunofluorescence staining were conducted to assess the influence of rh-sestrin2 on SAH-induced EBI and the underlying molecular pathway.

In the third experiment, Nrf2 inhibitor ML385 was employed to determine the role of Nrf2 signaling in the beneficial effects of rh-sestrin2 after SAH. Mice were randomly assigned into SAH, SAH treated with rh-sestrin2, SAH treated with rh-sestrin2 and ML385, and SAH treated with ML385 groups. Neurological function, ELISA, western blot analysis, and immunofluorescence staining were conducted. Animal groups, number of animals in each group, and mortality rates were shown in **Supplementary Table 1**.

### Drug administration

Recombinant human sestrin2 (rh-sestrin2) (Sigma-Aldrich) was dissolved in saline before use. Three different doses of rh-sestrin2 (1μg, 3μg, and 9μg) were administrated into the lateral ventricle at 2 h post-SAH. The doses of rh-sestrin2 was chosen based on an experimental brain ischemia study (18). ML385 (30 mg/kg) was given by intraperitoneally injection at 2 h before SAH as previously reported (25).

### Neurobehavioral tests

Functional deficiency was recorded at 24 and 72 h after surgery. A modified Garcia scale system was used to evaluate functional deficiency (26). The beam balance test was also employed in this study to assess motor dysfunction. In brief, mice were placed on a beam. The latency to fall and walking distance were recorded.

### Western blot analysis

Western blot analysis was processed as previously reported (27). The protein samples were placed into 10-12% gels and transferred to a PVDF membrane. The membrane was then supplemented with primary antibodies to sestrin2 (1:1000, 21346-1-AP, Proteintech), Nrf2 (1:1000, ab62352, Abcam), HO-1 (1:2000, ab68477, Abcam), NQO-1 (1:1000, ab34173, Abcam), β-actin (1:4000, AP0060, Bioworld), and Histone H3 (1:1000, 4499S, Cell Signaling Technology) at 4°C. After that, the membrane was supplemented with appropriate secondary antibodies. Bands were visualized with ECL reagent. The relative intensity was measured with ImageJ software.

### Immunofluorescence

Brain sections were fixed with 4% paraformaldehyde, and then permeabilized with TritonX-100 (0.5%). After that, they were blocked with 1% BSA, and then supplemented with primary antibodies to sestrin2 (1:100, 21346-1-AP, Proteintech), Nrf2 (1:100, ab31163, Abcam), 8-ohdg (1:100, ab62623, Abcam), Iba-1 (1:50, SC-98468, Santa Cruz; 1:100, ab178847, Abcam), CD16 (1:100, BD Biosciences), and CD206 (1:100, Invitrogen) at 4°C. The next day, the slices were incubated with the appropriate secondary antibodies, and were sealed with DAPI. The fluorescence intensity was measured with ImageJ software.

### TUNEL staining

TUNEL staining was conducted on frozen brain slices with a commercial TUNEL kit (Beyotime). In brief, brain slices were supplemented with the primary antibody anti-NeuN (1:200, EMD Millipore) at 4°C for one night. After that, they were incubated with TUNEL detection solution for 30 min. The TUNEL-positive neurons were recorded for statistical analysis.

### ELISA

The protein levels of IL-1β and TNF-α in brain samples were measured with commercial ELISA kits according to the manufacturer’s protocols (Multi Sciences Biotech). In brief, the tissues from the brain cortex were collected and homogenized. The supernatants from different groups were evaluated with different ELISA kits.

### Nissl staining

Nissl staining was conducted to reflect neuronal damage after SAH insults. As previously reported (28), brain slices were stained with cresyl violet solution. After three times washing with double-distilled water, the slices were mounted with permount.

### Statistical analysis

Data were summarized as mean ± SD. Statistical analysis was performed with the application of Prism V.9 software program (GraphPad Software, USA). One-way ANOVA followed by Tukey’s multiple comparisons test was performed to compare multiple groups. For neurological function, two-way ANOVA with Tukey’s multiple comparisons test was conducted. A value of *P* < 0.05 was considered statistically significant.

## Results

### Expression of sestrin2 in cortex after SAH

**Figure 1:**
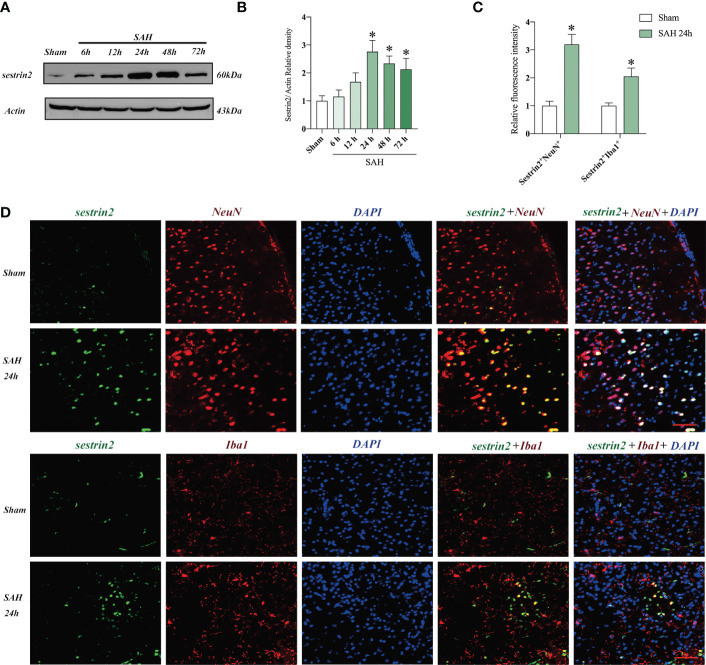
Expression and cellular distribution of sestrin2 in the early period after SAH. **(A)** Representative western blot bands and **(B)** quantitative analysis of sestrin2 in different groups (n = 6 per group). **(C)** Quantitative analysis of sestrin2 in neurons and microglia in sham and SAH groups (n = 6 per group). **(D)** Double immunofluorescence staining for sestrin2 in neurons and microglia in the brain cortex after SAH. ^*^ *P* < 0.05 versus sham group. Data indicated as mean ± SD. Scale bars = 50 μm.

The expression of sestrin2 in brain cortex after SAH remains unclear. In this study, we used western blot analysis and double immunofluorescence staining to assess endogenous sestrin2 expression in the early period after SAH. As shown in **Figures 1A, B**, the protein levels of sestrin2 was significantly increased at 12 h and peaked at 24 h after SAH as compared with sham group. The cellular distribution of sestrin2 was identified by double immunofluorescence staining. It showed that sestrin2 can be distributed in neurons and microglia, especially in neurons. SAH insults significantly induced sestrin2 expression in neurons when compared with sham group (**Figures 1C, D**).

### Effects of Rh-sestrin2 on neurological function and inflammatory response after SAH

**Figure 2:**
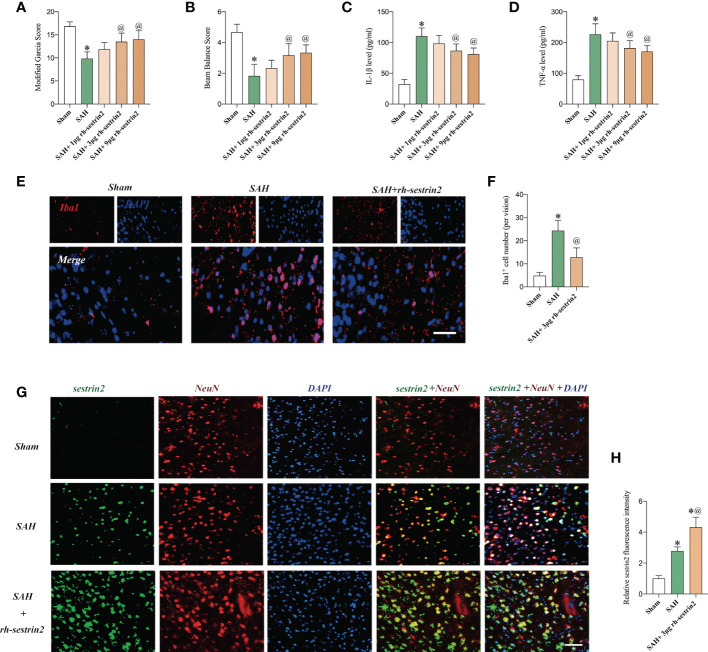
Rh-sestrin2 improved neurological function and reduced neuroinflammatory insults after SAH. **(A)** Modified Garcia score and **(B)** beam balance score indifferent experimental groups (n = 6 per group). Quantitative analyses of **(C)** IL-1β and **(D)** TNF-α in different experimental groups (n = 6 per group). **(E)** Representative immunofluorescence staining for Iba1. **(F)** Quantitative analysis of Iba1 staining (n = 6 per group). **(G)** Representative images and **(H)**quantifications of sestrin2 staining in different groups (n = 6 per group). *P < 0.05 versus sham group. @*P* < 0.05 versus SAH group. Data indicated asmean ± SD. Scale bars = 50 mm.

Sestrin2 is involved in modulation of multiple biological functions, such as immune response, oxidative insults, endoplasmic reticulum, ischemia, and hypoxia. More importantly, rh-sestrin2 has been shown to reduce neuroinflammation and improve neurological outcomes in a variety of CNS models. In this experiment, we then evaluated the effects of rh-sestrin2 on neurological function and inflammatory response after SAH. As shown (**Figures 2A-D**), both 3 μg and 9 μg rh-sestrin2 effectively improved neurological behavior and decreased pro-inflammatory cytokines release when compared with sham group. No statistical differences on neurological function and pro-inflammatory cytokines were detected between 3 μg and 9 μg rh-sestrin2 treatment. Our data also revealed that 3 μg rh-sestrin2 treatment significantly decreased microglia activation and increased sestrin2 expression in the cortex after SAH (**Figures 2E-H**).

### Rh-sestrin2 promoted M2 microglia polarization and decreased neuronal apoptosis after SAH

**Figure 3:**
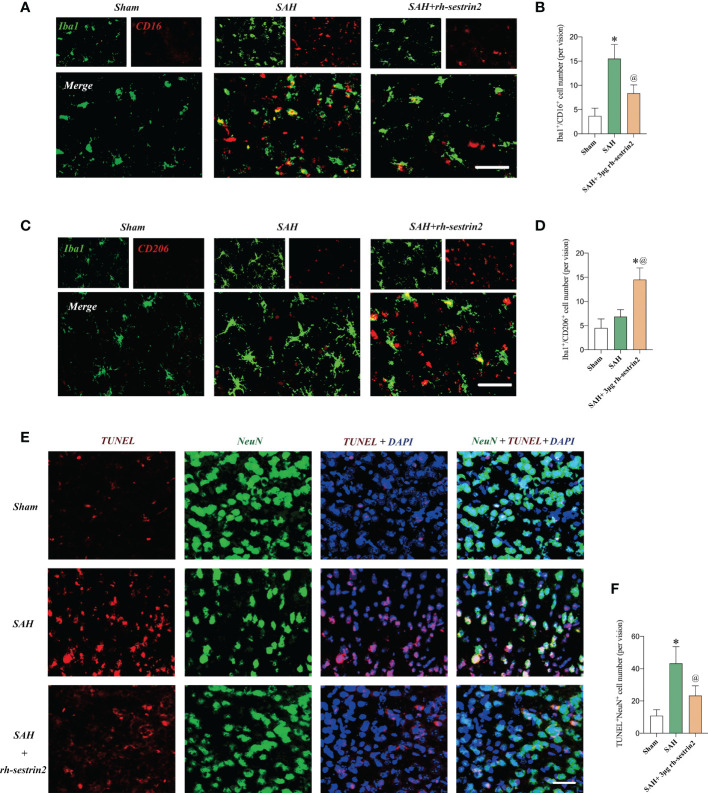
Rh-sestrin2 inhibited M1 microglia, increased M2 microglia polarization, and reduced neuronal apoptosis after SAH. **(A)** Representative images and **(B)** quantifications of CD16^+^/Iba1^+^ staining in different groups (n = 6 per group). **(C)** Representative images and **(D)** quantifications of CD206^+^/Iba1^+^ staining in different groups (n = 6 per group). **(E)** Representative images and **(F)** quantifications of TUNEL staining in different groups (n = 6 per group). ^*^ *P* < 0.05 versus sham group. ^@^ *P* < 0.05 versus SAH group. Data indicated as mean ± SD. Scale bars = 50 μm.

Recent studies indicate that sestrin2 could inhibit M1 microglia and promote M2 microglia polarization. However, the potential effects of rh-sestrin2 on microglia polarization after SAH remain obscure. Consistent with previous studies, the double immunofluorescence staining confirmed that SAH insults significantly increased the number of M1 microglia when compared to the sham group. However, SAH did not cause an evident increase in the number of M2 microglia as compared with the sham group. In contrast, rh-sestrin2 significantly decreased the number of M1 microglia (CD16^+^/Iba1^+^) and increased the number of M2 microglia (CD206^+^/Iba1^+^) after SAH (**Figures 3A-D**). Microglia polarization has been shown to play a vital role in neuronal apoptosis after SAH. We also investigated the effects of rh-sestrin2 on neuronal apoptosis. It showed that the number of TUNEL-positive neurons was dramatically increased after SAH, which could be significantly decreased after rh-sestrin2 (**Figures 3E, F**).

### Rh-sestrin2 induced Nrf2 signaling activation after SAH

**Figure 4:**
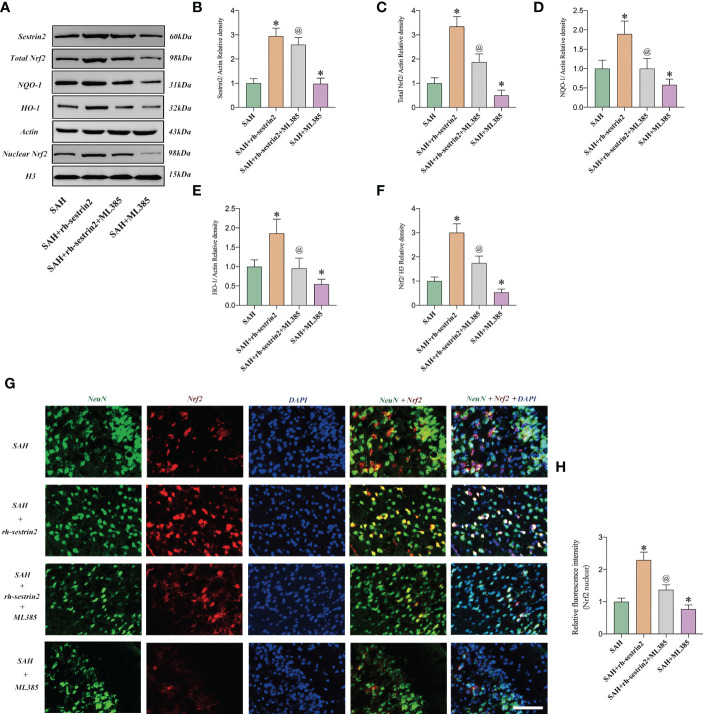
Rh-sestrin2 treatemtn induced Nrf2 signaling activation after SAH. **(A)** Representative western blot bands and quantitative analysis of **(B)** sestrin2, **(C)** total Nrf2, **(D)** NQO-1, **(E)** HO-1, and **(F)** nuclear Nrf2 in different groups (n = 6 per group). **(G)** Representative images and **(H)** quantifications of Nrf2 staining in different groups (n = 6 per group). ^*^ *P* < 0.05 versus sham group. ^@^ *P* < 0.05 versus SAH group. Data indicated as mean ± SD. Scale bars = 50 μm.

Mounting evidences has indicated that sestrin2 is a key upstream target of Nrf2. Targeting sestrin2 could significantly induce Nrf2 activation. We further evaluated the effects of rh-sestrin2 on Nrf2 signaling after SAH with the application of Nrf2 selective inhibitor ML385. Western blot analysis showed that rh-sestrin2 significantly increased Nrf2 expression, as well as the downstream targets including HO-1 and NQO-1. ML385, a selective Nrf2 inhibitor, was employed to suppress Nrf2 signaling. It showed that ML385 evidently reversed the effects of rh-sestrin2 on Nrf2 signaling pathway (**Figures 4A-F**). The double immunofluorescence staining verified that rh-sestrin2 markedly increased Nrf2 expression in nucleus after SAH, which could be abrogated by ML385 pretreatment (**Figures 4G, H**).

### ML385 reversed the anti-inflammatory and anti-oxidative effects of Rh-sestrin2 on SAH

**Figure 5:**
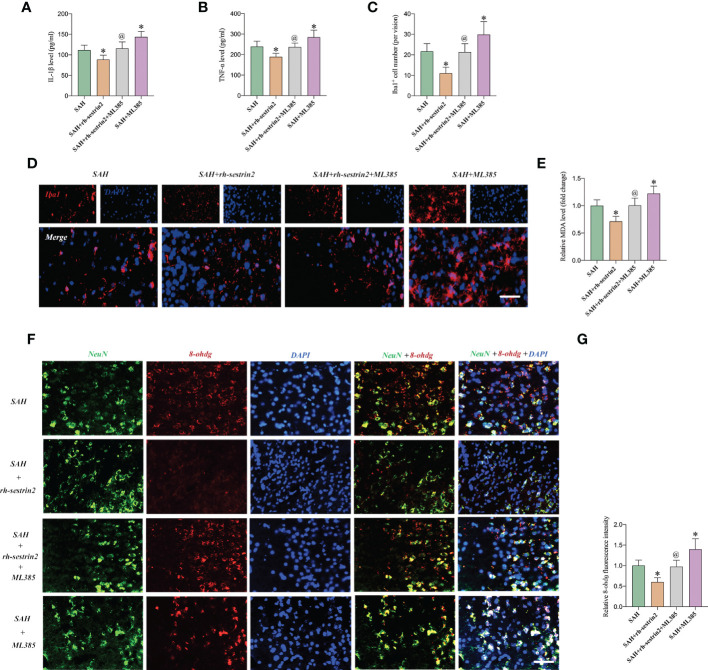
ML385 reversed the anti-inflammatory and anti-oxidative effects of rh-sestrin2 against SAH. Quantitative analyses of **(A)** IL-1β and **(B)** TNF-α in different experimental groups (n = 6 per group). **(C)** Quantitative analysis and **(D)** representative images of Iba1 staining in different groups (n = 6 per group). **(E)** Quantitative analyses of MDA (n = 6 per group). **(F)** Representative images and **(G)** quantifications of 8-ohdg staining in different groups (n = 6 per group). ^*^ *P* < 0.05 versus SAH group. ^@^ *P* < 0.05 versus SAH + rh- sestrin2 group. Data indicated as mean ± SD. Scale bars = 50 μm.

Based on our above-mentioned results, rh-sestrin2 could reduce inflammatory response after SAH. Nrf2 is a nuclear transcription factor and plays a crucial role in oxidative stress defense. We suspected that ML385 might reverse the anti-inflammatory and anti-oxidative effects of rh-sestrin2 on SAH. Our data showed that ML385 further exacerbated SAH-induced proinflammatory cytokines release and microglia activation when compared with SAH group. Meanwhile, ML385 abrogated the anti-inflammatory effects of rh-sestrin2 against SAH (**Figures 5A-D**). Moreover, rh-sestrin2 treatment could reduce oxidative damage and lipid peroxidation after SAH, which could be abated by ML385 (**Figures 5E-G**).

### ML385 reversed the effects of Rh-sestrin2 on microglia polarization after SAH

**Figure 6:**
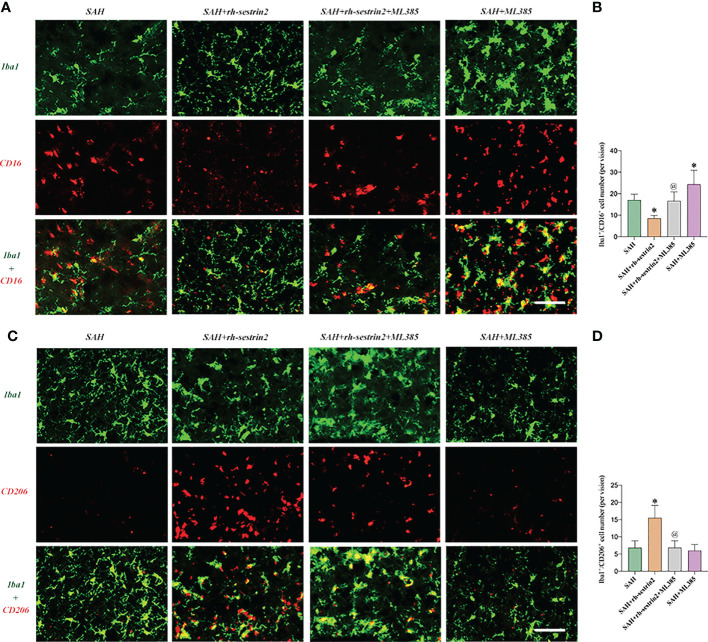
ML385 induced M1 microglia polarization and suppressed M2 microglia polarization after SAH. **(A)** Representative images and **(B)** quantifications of CD16^+^/Iba1^+^ staining in different groups (n = 6 per group). **(C)** Representative images and **(D)** quantifications of CD206^+^/Iba1^+^ staining in all groups (n = 6 per group). ^*^ *P* < 0.05 versus SAH group. ^@^ *P* < 0.05 versus SAH + rh- sestrin2 group. Data indicated as mean ± SD. Scale bars = 50 μm.

As mentioned above, rh-sestrin2 could suppress M1 microglia polarization and promote M2 microglia transformation. Nrf2 has been shown to modulate microglia polarization in other diseases models. We hypothesized that ML385 might reverse the effects of rh-sestrin2 on microglia polarization after SAH. The double immunofluorescence staining results confirmed that ML385 pretreatment further induced M1 microglia activation and decreased the number of M2 microglia after SAH. Meanwhile, the effects of rh-sestrin2 on microglia polarization were abrogated by Nrf2 suppression with ML385 (**Figures 6A-D**). These data suggested that rh-sestrin2 modulated M2 microglia polarization while suppressing M1 polarization after SAH *via* Nrf2-mediated signaling pathway.

### ML385 reversed the cerebroprotective effects of Rh-sestrin2 after SAH

**Figure 7:**
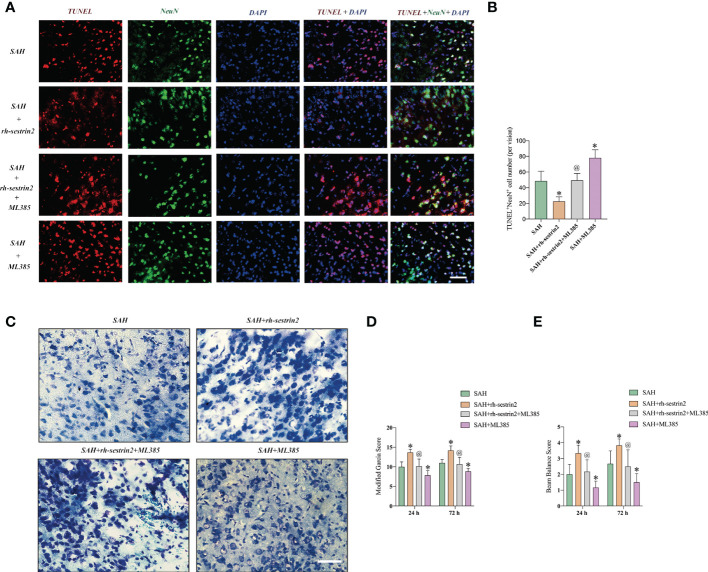
ML385 aggravated neuronal apoptosis and exacerbated neurological deterioration after SAH. **(A)** Representative images and **(B)** quantifications of TUNEL staining in different groups (n = 6 per group). **(C)** Representative images of Nissl staining in all groups. Quantifications of **(D)** modified Garcia score and **(E)** beam balance score (n = 6 per group). ^*^ *P* < 0.05 versus SAH group. ^@^ *P* < 0.05 versus SAH + rh- sestrin2 group. Data indicated as mean ± SD. Scale bars = 50 μm.

We then evaluated the effects of ML385 on neuronal apoptosis and neurological function after SAH. TUNEL staining showed that ML385 pretreatment further significantly aggravated neuronal apoptosis after SAH and reversed the anti-apoptotic effects of rh-sestrin2 on SAH. Meanwhile, rh-sestrin2 mitigated neuronal degeneration at day 3 after SAH, which could be abrogated after ML385 treatment (**Figures 7A-C**). Concomitant with the aggravated inflammatory insults and neuronal apoptosis, ML385 further exacerbated neurological deterioration and reversed the cerebroprotective effects of rh-sestrin2 against SAH (**Figures 7D, E**). Collectively, rh-sestrin2 could suppress SAH-induced inflammatory insults and ameliorate neurological dysfunction by promoting M2 microglia polarization and suppressing M1 microglia polarization *via* Nrf2 signaling pathway.

## Discussion

Mounting evidence has suggested that inflammatory insults is an independent predictor of unfavorable clinical outcomes in SAH patients (8, 29). After hemorrhage, microglia are activated promptly and their activation can persist for a long time. Under different stimulants, microglia can dynamically and temporally change their phenotype. M1 microglia has been demonstrated to induce amounts of proinflammatory mediators as well as ROS to exacerbate neuronal cell death and brain dysfunction. On the other hand, M2 microglia could clear toxic debris and release anti-inflammatory agents to resolve cerebrovascular inflammation (12). In a model of spinal cord injury, both M1 microglia and M2 microglia markers increased in the early period and microglia mainly polarized into M1 phenotype (30). In an experimental ischemic stroke, M1 microglia increased from day 3, whereas M2 microglia appeared on day 1 and persisted for 7 days (31). After experimental SAH, a mixed M1 and M2 microglia can be seen in brain cortex in the early period (32). Meanwhile, the microglial M1-to-M2 phenotype transition can be observed in the late stage of SAH (9). Thus, targeting the microglial phenotypic transformation after SAH might be a therapeutic approach.

Sestrin2 is a key component of the Sestrin family. Mounting evidence has indicated that sestrin2 could modulate different injuries, such as energy deficiency, oxidative insults, hypoxia stress, and immune response (33–35). In CNS diseases, sestrin2 has also been shown to exert neuroprotective effects. For example, Shi et al. reported that sestrin2 exerted anti-apoptosis effects, reduced brain atrophy, and improved function recovery after experimental hypoxic-ischemic encephalopathy (19). Sun et al. observed that sestrin2 deficiency exacerbated neuropathic pain behaviors and increased ROS production (17). In models of TBI, sestrin2 overexpression also decreased neuronal apoptosis, oxidative insults, and neurological deficit (22). However, the potential role of sestrin2 on SAH-induced brain injury remains unknown. In the present study, we measured the time-course of sestrin2 in the brain tissue after SAH. We found that sestrin2 levels markedly increased and peaked around day 1 after SAH. This result was consistent with a previous report which illustrated that sestrin2 expression increased significantly in the early period after cerebral ischemia (16). At the cellular level, sestrin2 is primarily distributed in neurons (17). Similarly, our data revealed that the elevated sestrin2 after SAH was mainly distributed in neurons. Whether sestrin2 exerts a role in modulating inflammatory response, especially microglia polarization, is still obscure.

Recent studies have observed that sestrin2 participates in modulating microglia-mediated inflammatory response. Sun et al. reported that sestrin2 overexpression inhibited astrocytes and microglia activation, decreased the production of proinflammatory cytokines release, and restored mitochondrial biogenesis in the spinal cord of a rat model of osteoarthritis pain (17). In experimental cerebral ischemia, He et al. demonstrated that sestrin2 mitigated infarction volume, reduced neuronal apoptosis, and improved neurological function. The cerebroprotective effects of sestrin2 was associated with its anti-inflammatory effects by suppressing M1 microglia and promoting M2 microglia polarization (18). In parallel with these studies, our experiments revealed that sestrin2 overexpression significantly mitigated SAH-induced inflammatory insults by suppressing microglia M1 polarization and polarizing microglia from M1 to M2 phenotype. Concomitant with the decreased inflammatory insults, sestrin2 overexpression reduced neuronal apoptosis and ameliorated neurological dysfunction after SAH.

How sestrin2 regulates microglia polarization remains unclear. Nrf2 is a nuclear transcription factor and plays a crucial role in oxidative stress defense (36, 37). More importantly, in the CNS, Nrf2 has been shown to regulate microglia polarization (38–40). Hu et al. reported that Nrf2 activator omaveloxolone inhibited M1 microglia activation, promoted M2 microglial polarization, and ameliorated secondary brain insults after experimental intracerebral hemorrhage (ICH) both *in vivo* and *in vitro*. Nrf2 deletion abrogated the anti-inflammatory effects of omaveloxolone on ICH (38). Another study indicated that magnolol exhibited anti-depressant effects and promoted M2 microglia polarization by activation of Nrf2 signaling. Nrf2 deficiency abolished the magnolol-mediated microglia polarization (39). Interestingly, a great deal of research has indicated that sestrin2 is a key upstream target of Nrf2 (15, 22). Targeting sestrin2 could significantly induce Nrf2 activation in a variety of diseases models. We hypothesized that sestrin2 overexpression might modulate Nrf2 signaling to mediate microglia polarization after SAH. We then evaluated the expression of Nrf2 after sestrin2 overexpression. As expected, our experiment revealed that sestrin2 overexpression induced Nrf2 expression and further reduced oxidative damage after SAH. By using a highly selective Nrf2 inhibitor ML385, the relationship between sestrin2 and Nrf2 after SAH was verified. We observed that ML385 significantly suppressed Nrf2 activation by sestrin2 overexpression. Moreover, sestrin2-mediated microglia polarization and its anti-inflammatory effects were abrogated by ML385 treatment. Meanwhile, Nrf2 deficiency by ML385 further exacerbated neuronal apoptosis and neurological deficits after SAH. Our study demonstrated that sestrin2 could inhibit inflammatory insults and promote M2 microglia polarization by increasing Nrf2 expression.

It should be noted that sestrin2 modulated microglia polarization might not be dependent solely on Nrf2. Peroxisome proliferator-activated receptor coactivator-1α (PGC-1α), a transcription co-activator for nuclear receptors, has been shown to suppress M1 microglia activation and promote M2 microglia polarization in many CNS disorders (41). More importantly, PGC-1α plays a key role in neuronal survival after SAH (42). Sestrin2 could induce PGC-1α activation to facilitate cancer cell survival (43). Besides, some previous studies indicated that inhibiting NLRP3 after SAH could drive the microglial phenotype toward M2 (32). Sestrin2 has also been shown to protect against NLRP3-meidaited pyroptosis in many diseases models (35, 44). However, we did not evaluate the potential influence of sestrin2 on these molecular targets. Additional studies are further required to elucidate these issues.

## Conclusions

In conclusion, we provided the first preclinical evidence that sestrin2 mitigated SAH-induced EBI by inhibiting inflammatory insults. Sestrin2 overexpression could suppress microglia M1 polarization and promote microglia polarization to M2 by inducing Nrf2 signaling. Collectively, our findings indicated that sestrin2 might be a potential new target for treating SAH insults.

## Data availability statement

The original contributions presented in the study are included in the article/**Supplementary Material**. Further inquiries can be directed to the corresponding authors.

## Ethics statement

The animal study was reviewed and approved by The Ethics Committee of The First Affiliated Hospital of Nanchang University.

## Author contributions

YY, TH, XWa, and XWu contributed to the design of study. YY, HD, CY, JW, YB, SL, LiZ, LuZ, and BL conducted the *in vivo* experiments. YY wrote the manuscript. All authors contributed to the article and approved the submitted version.
