# Supplementary material for: Sestrin2 provides cerebral protection through activation of Nrf2 signaling in microglia following subarachnoid hemorrhage
Source: Front Immunol. 2023 Jan 24;14:1089576. doi: 10.3389/fimmu.2023.1089576 (PMC9903076; doi:10.3389/fimmu.2023.1089576)
Supplement: Supplementary file 1 [file Table_1.docx]

Supplementary Table 1. Animal groups and mortality rates

| Group | Alive | Dead | Mortality rate |
| --- | --- | --- | --- |
| Sham | 18 | 0 | 0 |
| SAH | 71 | 11 | 15.5% |
| SAH + 1μg rh-sestrin2 | 7 | 1 | 14.3% |
| SAH + 3μg rh-sestrin2 | 35 | 5 | 14.3% |
| SAH + 9μg rh-sestrin2 | 7 | 1 | 14.3% |
| SAH + 3μg rh-sestrin2 + ML385 | 22 | 4 | 18.2% |
| SAH + ML385 | 23 | 5 | 21.7% |
